# A new combination in *Lapanthus* (Bromeliaceae)

**DOI:** 10.3897/phytokeys.17.3642

**Published:** 2012-10-26

**Authors:** Rafael Batista Louzada, Maria das Graças Lapa Wanderley

**Affiliations:** 1 Departamento de Botânica, Instituto de Biociências, Universidade de São Paulo, São Paulo, SP, Brazil; 2 Instituto de Botânica, Secretaria do Meio Ambiente, 01061–970, São Paulo, SP, Brazil

**Keywords:** Bromelioideae, *Cryptanthus*, Espinhaço Range, Minas Gerais, *Orthophytum*

## Abstract

A new combination, *Lapanthus vidaliorum* (O.B.C. Ribeiro & C.C. Paula) Louzada & Wand. is proposed for *Orthophytum vidaliorum* O.B.C. Ribeiro & C.C. Paula. In addition notes on taxonomy, geographic distribution and conservation are provided.

## Introduction

*Lapanthus* Louzada & Versieux is a small genus comprising two species occurring in the southern portion of the Espinhaço Range in the Brazilian state of Minas Gerais. The species inhabits quartzitic rocky outcrops near waterfalls and gallery forests in rocky fields or areas of transitional vegetation between semideciduous seasonal forests and rocky fields (Louzada and Versieux 2010).

*Lapanthus* was established to accommodate two species, one previously included in *Orthophytum* Beer and the other in *Cryptanthus* Otto & A. Dietr. (Louzada and Versieux 2010). The two originally recognized species of *Lapanthus* (*Lapanthus duartei* (L.B. Sm.) Louzada & Versieux and *Lapanthus itambensis* (Versieux & Leme) Louzada & Versieux)were segregated from *Cryptanthus* and *Orthophytum* respectively due to the presence of characters apparently contradictory to the current circumscriptions of those genera, which include ciliate petal margins, presence of a pair of lanceolate petal appendages, and free stamens (Louzada and Versieux 2010). The decision to describe a new genus to accommodate the species with these characters was also supported by the evidence of paraphyletism of *Orthophytum* presented in the molecular phylogeny of Bromelioideae (Schulte et al. 2009). In this study, *Orthophytum supthutii* E. Gross & Barthlott, recently synonymized under *Lapanthus duartei*, arises as the sister group of a clade including *Cryptanthus glaziovii* Mez, *Orthophytum disjunctum* L.B. Sm. and *Orthophytum maracasense* L.B. Sm.

The phylogenetic relationship of the genus is further elucidated by a study on molecular phylogeny where *Lapanthus* arises as a monophyletic group, sister to a *Cryptanthus* clade comprising species of *Cryptanthus* subgen. *Cryptanthus* (Louzada et al. in prep.).

**Table 1.** Comparison of some diagnostic characters of *Lapanthus*, *Cryptanthus* and *Orthophytum*.

Character	*Lapanthus*	*Cryptanthus*	*Orthophytum*
Inflorescence	sessile	sessile	pedunculate or sessile
Sepals	white	green	green or red
Petal margins	ciliate	entire or ciliate	entire
Petal appendages type	lanceolate	absent	sacciform, cupuliform or fimbriate
Antepetalous stamens	free	adnate, rarely free	adnate
Epigynous tube	absent	absent or short	present
Meiotic chromosome number	*n* = 50	*n* = 17	*n* = 25
Mitotic chromosome number	2*n* = 50	2*n* = 34, 36, 54	2*n* = 50, 100, 150

## Taxonomy

### Lapanthus vidaliorum

(O.B.C. Ribeiro & C.C. Paula) Louzada & Wand. comb. nov.

urn:lsid:ipni.org:names:77122665-1

http://species-id.net/wiki/Lapanthus_vidaliorum

Figs 1, A–B , 2

Orthophytum vidaliorum O.B.C. Ribeiro & C.C. Paula. Brittonia 62: 145, f. 1. 2010. Type: Brazil. Minas Gerais: Santa Bárbara, Serra de Capanema, 20°11'29"S, 43°35'05.1"W, 1469 m elev., 19 Aug 2008, *O.B.C. Ribeiro 208* (holotype: VIC!; isotype: HB). [Basionym]

#### Notes.

When *Orthophytum vidaliorum* was described, Ribeiro and Paula (2010) discussed its morphological relationship with *Orthophytum itambense*. It was emphasized that these species share similar habitat, plant size, leaves, inflorescence and flower structure. Moreover, they state that *Orthophytum vidaliorum* is also closely related to *Orthophytum supthutii* which was recently synonymized under *Lapanthus duartei*. In the same article the authors also mentioned that *Orthophytum vidaliorum* could be included in a different and unpublished genus proposed by Louzada (2008) in his master’s thesis, which later was validly published under the name *Lapanthus* by Louzada and Versieux (2010).

Besides, some morphological characters such as the rosette shape, the morphology and size of the leaves and the size of the flowers support the decision to include *Orthophytum vidaliorum* under *Lapanthus*.

**Table 2.** Comparison of diagnostic characters in *Lapanthus* species.

Character	*Lapanthus vidaliorum*	*Lapanthus duartei*	*Lapanthus itambensis*
Leaf-blade indument	glabrous	lepidote	lepidote
Inflorescence branching	simple	compound	pseudo-simple
Petal length	2.5–2.6 mm	2.8–3.8 mm	4.1 mm
Petal color	greenish-yellow	orange	white
Petal appendages	obdeltoid	lanceolate	lanceolate

**Identification key for the species of *Lapanthus***

**Table:** 

1a	Inflorescence compound, sepals high connate	*Lapanthus duartei*
1b	Inflorescence simple or pseudo-simple, sepals free or nearly so	2
2a	Inflorescence simple, petals greenish-yellow, petal appendages obdeltoid	*Lapanthus vidaliorum*
2b	Inflorescence pseudo-simple, petals white, petal appendages lanceolate	*Lapanthus itambensis*

**Figure 1.**
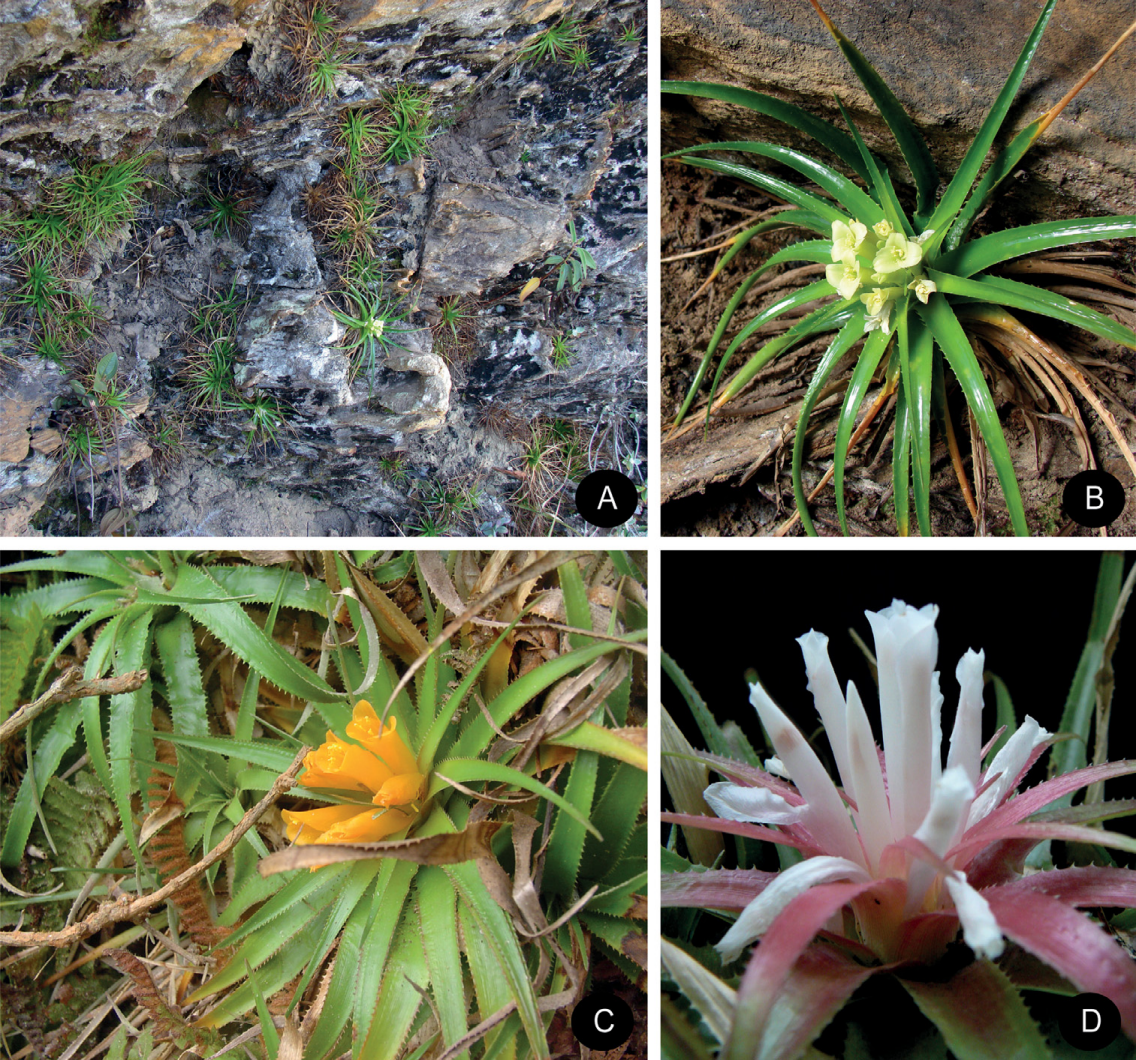
**A–B**. *Lapanthus vidaliorum*. **A** Habitat **B** Habitat in the wild **C** *Lapanthus duartei* in the wild **D** *Lapanthus itambensis* in cultivation (Photo: **A–B** Otávio Ribeiro).

**Figure 2.**
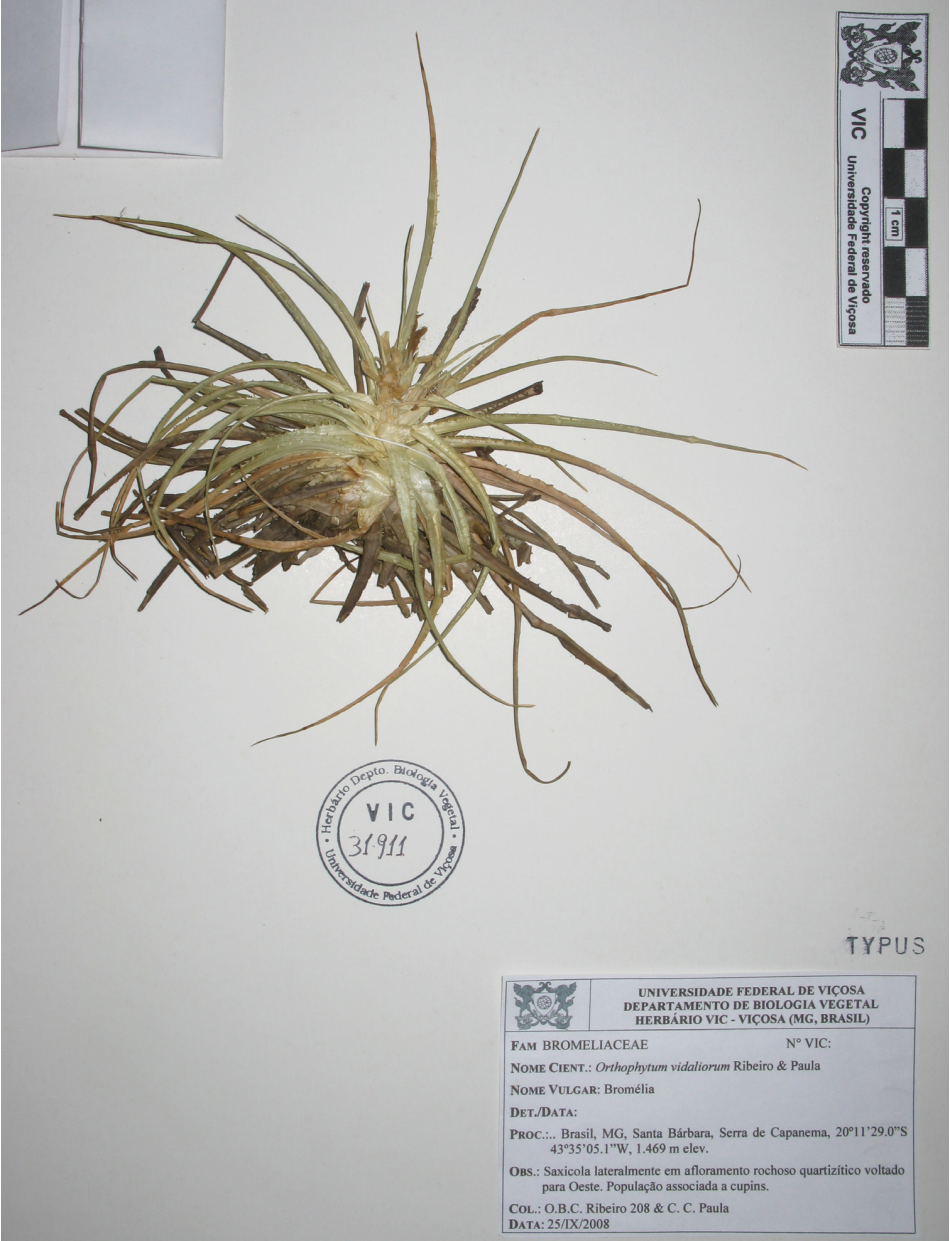
Holotype of *Lapanthus vidaliorum* (Photo: Elídio Guarçoni).

#### Distribution.

**Figure 3.**
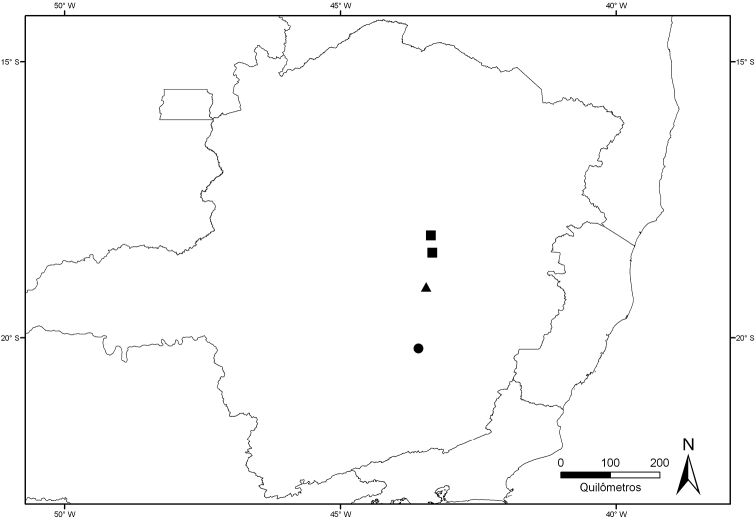
Distribution map of *Lapanthus vidaliorum* (circle), *Lapanthus duartei* (triangle), and *Lapanthus itambensis* (square).

*Lapanthus vidaliorum* occurs in the southernmost part of the Espinhaço Range, in an iron–rich region called Quadrilátero Ferrífero (Iron Quadrangle) in the Brazilian state of Minas Gerais. Although it occurs in an iron–rich area, *Lapanthus vidaliorum* was found inhabiting quartizitic–sandstone rocky outcrops (Ribeiro and Paula 2010) The present combination extends the genus distribution approximately 120 km southward (Fig. 3).

#### Conservation.

*Lapanthus vidaliorum* is an endangered species, known only from the type–population, which is small in number of individuals, being about 3.5 kilometers from the iron ore mine Capanema and surrounded by an *Eucalyptus* plantation. Therefore, according to IUCN (2001) criteria this species is considered critically endangered (criteria B2a).

## Supplementary Material

XML Treatment for Lapanthus vidaliorum
